# Peptidoglycan in obligate intracellular bacteria

**DOI:** 10.1111/mmi.13880

**Published:** 2017-12-12

**Authors:** Christian Otten, Matteo Brilli, Waldemar Vollmer, Patrick H. Viollier, Jeanne Salje

**Affiliations:** ^1^ The Centre for Bacterial Cell Biology Institute for Cell and Molecular Biosciences, Newcastle University Newcastle upon Tyne NE2 4AX UK; ^2^ Department of Agronomy, Food, Natural Resources, Animals and Environment (DAFNAE) University of Padova. Agripolis ‐ V.le dell'Università, 16 | 35020 Legnaro Padova Italy; ^3^ Department of Microbiology and Molecular Medicine Institute of Genetics & Genomics in Geneva (iGE3), University of Geneva Geneva Switzerland; ^4^ Nuffield Department of Medicine, Centre for Tropical Medicine and Global Health University of Oxford Oxford UK; ^5^ Mahidol‐Oxford Tropical Medicine Research Unit Mahidol University Bangkok Thailand; ^6^ Present address: Department of Biosciences University of Milan, via Celoria 26 (MI) Italy

## Abstract

Peptidoglycan is the predominant stress‐bearing structure in the cell envelope of most bacteria, and also a potent stimulator of the eukaryotic immune system. Obligate intracellular bacteria replicate exclusively within the interior of living cells, an osmotically protected niche. Under these conditions peptidoglycan is not necessarily needed to maintain the integrity of the bacterial cell. Moreover, the presence of peptidoglycan puts bacteria at risk of detection and destruction by host peptidoglycan recognition factors and downstream effectors. This has resulted in a selective pressure and opportunity to reduce the levels of peptidoglycan. In this review we have analysed the occurrence of genes involved in peptidoglycan metabolism across the major obligate intracellular bacterial species. From this comparative analysis, we have identified a group of predicted ‘peptidoglycan‐intermediate’ organisms that includes the Chlamydiae, *Orientia tsutsugamushi, Wolbachia* and *Anaplasma marginale*. This grouping is likely to reflect biological differences in their infection cycle compared with peptidoglycan‐negative obligate intracellular bacteria such as *Ehrlichia* and *Anaplasma phagocytophilum*, as well as obligate intracellular bacteria with classical peptidoglycan such as *Coxiella, Buchnera* and members of the *Rickettsia* genus. The signature gene set of the peptidoglycan‐intermediate group reveals insights into minimal enzymatic requirements for building a peptidoglycan‐like sacculus and/or division septum.

## Introduction

### Peptidoglycan structure and function

Peptidoglycan (also called murein) is one of the largest macromolecules in a bacterial cell, typically forming a mesh‐like structure called the peptidoglycan sacculus that encases the cytoplasmic membrane (Vollmer *et al*., 2008a; Weidel and Pelzer, 1964). In Gram‐negative (or, more precisely, diderm) bacteria, this peptidoglycan sacculus resides in the periplasm between the cytoplasmic and outer membrane, whilst in Gram‐positive (monoderm) species the peptidoglycan layer is thicker and connected with other major cell wall polymers such as wall teichoic acid, capsular polysaccharide and the S‐layer (Weidenmaier and Peschel, 2008; Silhavy *et al*., 2010). Peptidoglycan is structurally distinct from cell wall components in archaea and single celled eukaryotes (with the exception of certain plant and algae chloroplasts), and has no homolog in multicellular eukaryotic organisms. Peptidoglycan has at least three major functions. First, it enables the bacterial cell to sustain the high turgor, which results from the difference between the high osmolarity of the bacterial cytoplasm and the comparatively low osmolarity of the external environment. Second, peptidoglycan maintains the shape of a bacterial cell. Third, it provides rigidity to envelope‐spanning surface structures such as flagella and retractile pili that exert force and require a solid support to push or pull against. The essentiality of peptidoglycan for survival of bacteria in a hypoosmolar environment along with its role in anchoring cell surface appendages that are often important virulence determinants makes it an attractive antibiotic target, and multiple classes of clinically successful antibiotics target various aspects of peptidoglycan synthesis, for example beta‐lactams and glycopeptides (Silver, 2013).

**Figure 1:**
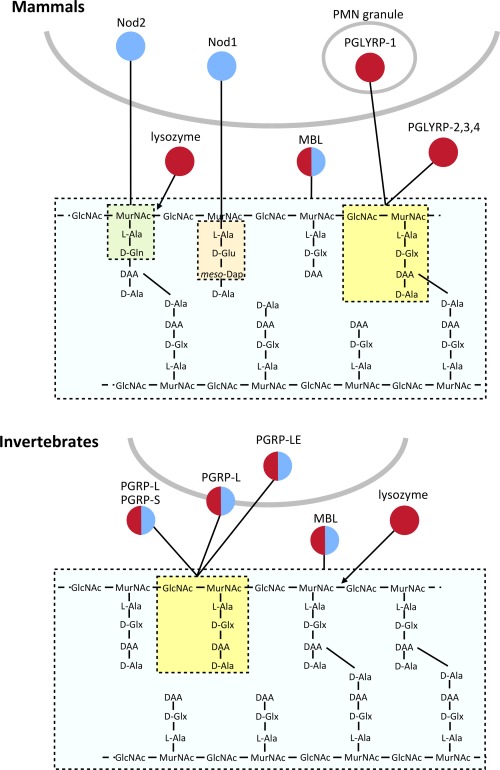
Summary of peptidoglycan recognition proteins in invertebrates and mammals. An overview of peptidoglycan recognition proteins in invertebrates and mammals. Some proteins degrade peptidoglycan (shown in red) whilst others induce downstream signalling pathways (shown in blue). Polymerised peptidoglycan is shown, with fragments recognised by different PGRPs indicated by dotted lines/boxes.

Peptidoglycan is composed of polysaccharide chains made up of alternating ß‐1,4‐linked N‐acetylglucosamine (Glc*N*Ac) and N‐acetylmuramic acid (Mur*N*Ac) residues which are connected via short peptides (Fig. 1) (Weidel and Pelzer, 1964; Vollmer *et al*., 2008a). These peptides contain D‐amino acids such as D‐alanine or D‐glutamate, as well as unusual non‐proteinogenic amino acids, such as *meso‐*diaminopimelic acid (*meso*‐DAP). The length of individual glycan chains, the amino acid sequence of the peptides and the structure of cross‐links are variable between species and may differ at distinct stages of growth in one species (Vollmer and Höltje, 2004; Vollmer, 2008; Vollmer and Seligman, 2010). Chemical modifications are found in the glycan backbone or peptides, and these may have emerged in response to selective pressure on peptidoglycan from peptidoglycan‐targeting enzymes or antibiotics (Vollmer and Tomasz, 2002; Vollmer, 2008; Figueiredo *et al*., 2012). Given the importance of a structurally intact sacculus on bacterial cell integrity, the polymerisation and insertion of new peptidoglycan strands is a complex and robustly regulated process (Typas *et al*., 2012; Pazos *et al*., 2017) and this is particularly critical in the context of bacterial cell division (Egan and Vollmer, 2013).

### Immune responses to peptidoglycan

The innate immune response of vertebrates and invertebrates has evolved a repertoire of components to detect and destroy invading bacteria. This is achieved through recognition of characteristic non‐self structures, called pathogen associated molecular patterns (PAMPs). Peptidoglycan is an important PAMP, due to its presence in virtually all bacteria and its almost complete absence in higher eukaryotes. Specific fragments released from peptidoglycan are recognised by various peptidoglycan recognition proteins (Fig. 1 and Table 1) (Sukhithasri *et al*., 2013; Neyen and Lemaitre, 2016). The sacculus is hidden from the innate immune system by the presence of an outer membrane in intact diderm bacteria, but soluble peptidoglycan turnover products can be released from intact cells either directly into the surrounding milieu or within outer membrane vesicles. Furthermore, bacterial cells undergoing lysis release peptidoglycan fragments into the extracellular environment. (Goodell and Schwarz, 1985; Uehara and Park, 2008; Schwechheimer *et al*., 2013). Some peptidoglycan recognition proteins directly hydrolyse peptidoglycan through their amidase or muramidase activity, whilst others have no enzymatic activity but activate a downstream signaling pathway in response to binding peptidoglycan fragments which can then lead to immune cell maturation or the release of proinflammatory cytokines (Fig. 1) (Boneca, 2005; Chaput and Boneca, 2007). Whilst the aim of this immune response is to clear the bacterial infection, overstimulation of the inflammatory response can lead to fatal conditions such as septic shock (Calandra, 2001; Neyen and Lemaitre, 2016). Peptidoglycan recognition proteins are located in multiple organs and tissues throughout the body, and can be located extracellularly, intracellularly or attached to the surface of a host cell (Girardin and Philpott, 2004; Royet and Dziarski, 2007; Dziarski and Gupta, 2010). This ensures the detection of invading pathogens in almost every possible location (Fig. 1 and Table 1).

**Table 1:** Overview of peptidoglycan recognition proteins in mammals and invertebrates

Peptidoglycan recognition protein	Peptidoglycan fragment detected	Main effect	Main tissue distribution	Cellular localisation
**MAMMALS (*H. sapiens)***				
PGLYRP 2	GlcNAc‐MurNAc‐tetrapeptide	Amidase activity	Liver, skin, oral, intestinal	Soluble
PGLYRP 1,3,4	GlcNAc‐MurNAc‐tetrapeptide	Bactericidal	PMN granules, skin, sweat glands, sebaceous glands, mouth, intestinal tract, eyes	Soluble (PGLYRP 3,4), PMN granules (PGLYRP 1)
Nod1	Tripeptide: L‐Ala‐D‐Glu‐DAP	Activation of pro‐inflammatory pathway via NF‐κB signalling	Ubiquitous	Cytoplasm
Nod2	GlcNAc‐MurNAc‐Ala‐Glu	Activation of pro‐inflammatory pathway via NF‐κB signalling	Monocytes	Cytoplasm
Lysozyme	MurNAc‐GlcNAc glycosidic bond	Muramidase activity; bactericidal effects	Phagocytic granules, serum, body secretions	Soluble
C‐type lectins (e.g. MBL, RegIII)	Glycan polymer	Complement activation; bactericidal effects	Serum	Soluble
**INVERTEBRATES *(D. melanogaster)***				
PGRP‐L (multiple)	GlcNAc‐MurNAc‐tetrapeptide (Lys‐type)	Amidase activity; induction of antimicrobial peptides via activation of *Imd* pathway; phagocytosis	Haemocytes, fat body, gut, trachea, haemolymph	Cell surface, cytoplasm and soluble
PGRP‐S (multiple)	GlcNAc‐MurNAc‐tetrapeptide (DAP‐type)	Amidase activity; induction of antimicrobial peptides via activation of Toll pathway; phagocytosis; general bactericidal activity	Haemocytes, fat body, gut, trachea, epidermis, haemolymph	Soluble
Lysozyme	MurNAc‐GlcNAc glycosidic bond	Muramidase activity; general bactericidal activity	Gut, salivary glands	Soluble
C‐type lectins	Glycan polymer	Encapsulation; melanisation; bactericidal activity	Haemolymph, fat body	Cell surface and soluble

### Obligate intracellular bacteria

**Figure 2:**
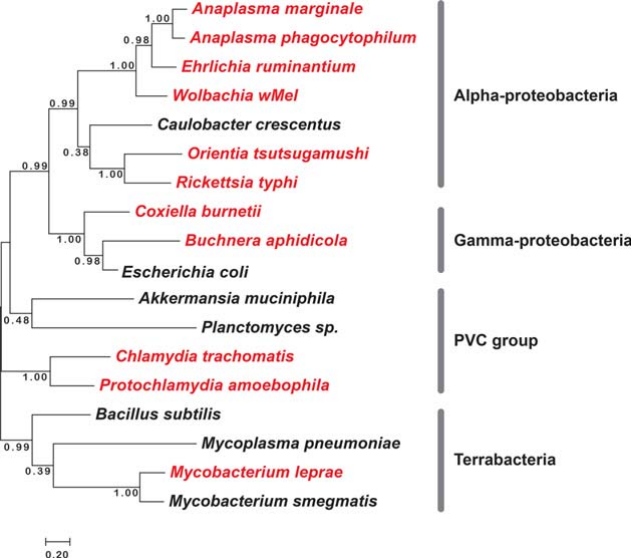
Phylogenetic tree showing relationship between selected obligate intracellular and free‐living bacteria discussed in this review. Obligate intracellular bacteria are shown in red, and free‐living bacteria are shown in black.

Bacterial species have evolved to exploit an enormous diversity of environmental niches for their growth and replication. One of the most specialised replicative niches is the interior of a living eukaryotic cell. Some bacterial pathogens adopt this niche at some point during their lifecycle (facultative intracellular bacteria) whilst others have become so adapted to this environment that they have lost the ability to replicate in the absence of their cellular host (obligate intracellular bacteria). These intracellular bacteria typically replicate either free in the eukaryotic cytoplasm, or within specialised vacuoles. It is hypothesised that an early mutualistic adaptation between bacteria and archaea led to the emergence of modern mitochondria and chloroplasts (Bonen *et al*., 1977; Kuntzel *et al*., 1981; Carvalho *et al*., 2015). The major groups of obligate intracellular bacteria are the phylogenetically distinct Chlamydiales and Rickettsiales orders, as well as *Coxiella, Buchnera* and *Mycobacterium leprae* (Fig. 2 and Table 2). Whilst most cause human or animal disease, a small number (including *Wolbachia, Buchnera* and *Protochlamydia amoebophila)* are not known to cause disease and have a mutualistic relationship with their eukaryotic hosts. A common feature of most diverse species of obligate intracellular bacteria is their significantly reduced genome (compared to most free‐living bacteria), which is a consequence of the adaptation to the stable, intracellular environment and of their parasitic lifestyle. An overview of representative obligate intracellular bacteria, their pathogenesis and their cellular lifestyles, is given in Table 2.

**Table 2:** Lifestyle and pathogenesis of selected obligate intracellular bacteria

LPS (LpxA)	Peptidoglycan (Class A PBPs)	Peptidoglycan (Class B PBPs)	Bacteria	Human disease	Animal reservoir(s)	Vector/ spread	Cellular niche	Major cellular tropism (in human infections)	Primary tissue tropism (in human infections)
CHLAMYDIALES									
**+**	**–**	**+**	*Chlamydia pneumoniae*	Pneumonia	–	Aerosol	Vacuole	Epithelial cells, endothelial cells, macrophages	Lungs, heart
**+**	**–**	**+**	*Chlamydia trachomatis*	Urethritis, pneumonia, trachoma	–	Direct contact	Vacuole	Epithelial cells, monocytes/ macrophages	Genitourinary tract, lungs, eyes
**+**	**–**	**+**	*Waddlia chondrophila*	Associated with miscarriage	–	Unknown	Vacuole	Placental cells	Placenta
**+**	**–**	**+**	*Simkania negevensis*	Pneumonia	–	Unknown	Vacuole	Epithelial cells	Respiratory tract
**+**	**–**	**+**	*Protochlamydia amoebophila*	–	–	Amoeba	Vacuole	–	–
RICKETTSIALES									
**–**	**–**	**+**	*Orientia tsutsugamushi*	Scrub typhus	Rodents	Mite	Cytoplasm	Endothelial cells, dendritic cells, monocytes/macrophages	Vascular endothelium
**+**	**+**	**+**	*Rickettsia prowazekii*	Epidemic typhus and Brill‐Zinsser disease	Flying squirrels	Louse	Cytoplasm	Endothelial cells	Vascular endothelium
**+**	**+**	**+**	*Rickettsia typhi*	Murine typhus	Rats	Flea	Cytoplasm	Endothelial cells	Vascular endothelium
**+**	**+**	**+**	*Rickettsia rickettsii*	Rocky mountain spotted fever	Dogs, rabbits, birds	Tick	Cytoplasm	Endothelial cells	Vascular endothelium
**+**	**+**	**+**	*Rickettsia akari*	Rickettsialpox	House mice, rats	Mite	Cytoplasm	Monocytes/macrophages	Blood
**+**	**+**	**+**	*Rickettsia conorii*	Mediterranean spotted fever	Rodents, dogs	Tick	Cytoplasm	Endothelial cells	Vascular endothelium
**–**	**–**	**+**	*Anaplasma marginale*	**–**	Cattle, wild ruminants	Tick	Vacuole	Erythrocytes	Blood
**–**	**–**	**–**	*Anaplasma phagocytophilum*	Human granulocytic anaplasmosis (HGE)	Deer, cats, dogs, ruminants, rodents	Tick	Vacuole	Neutrophils	Blood
**–**	**–**	**–**	*Ehrlichia chaffeensis*	Human monocytic ehrlichiosis (HME)	Dogs, deer	Tick	Vacuole	Monocytes/macrophages, endothelial cells	Blood, vascular endothelium
**–**	**–**	**–**	*Ehrlichia ruminatum*	–	Cattle, wild ruminants	Tick	Vacuole	Monocytes/macrophages, endothelial cells	Blood, vascular endothelium
**–**	**–**	**–**	*Neorickettsia sennetsu*	Sennetsu fever	Fish	Trematode	Vacuole	Monocytes/macrophages	Blood
**–**	**–**	**+**	*Wolbachia WMel*	–	–	Arthropod, insect, nematode	Vacuole	–	–
OTHER									
**+**	**+**	**+**	*Coxiella burnettii*	Q fever	Ruminants	Tick and aerosol	Vacuole	Monocytes/macrophages	Liver, lungs, heart
**–**	**+**	**+**	*Buchnera aphidicola*	**–**	–	Pea aphid	Vacuole	–	–
**–**	**+**	**+**	*Mycobacterium leprae*	Leprosy	Armadillos	Aerosol	Vacuole	Histiocytes, nerve cells, macrophages, epithelial cells	Skin

**Table 3:** Summary of all genes included in this study

Gene name	KEGG number	Protein function
*alr*	K01775	Alanine racemase
*amiA,B,C*	K01448	N‐Acetylmuramoyl‐L‐alanine amidase
*amiD*	K11066	N‐Acetylmuramoyl‐L‐alanine amidase
*ampG*	K08218	MFS transporter, PAT family, beta‐lactamase induction signal transducer
*ampH*	K18988	Serine‐type D‐Ala‐D‐Ala carboxypeptidase/endopeptidase
*anmK*	K09001	Anhydro‐N‐acetylmuramic acid kinase
*argD*	K00821	Acetylornithine/N‐succinyldiaminopimelate aminotransferase
*asd*	K00133	Aspartate‐semialdehyde dehydrogenase
*aspC*	K10206	Aspartate aminotransferase
*bacA (uppP)*	K06153	Undecaprenyl‐diphosphatase
*dacA*	K01286	DD‐Carboxypeptidase PBP5
*dacB*	K07259	DD‐Carboxy‐/endopeptidase PBP4
*dacC*	K07258	D‐Alanyl‐D‐alanine carboxypeptidase; penicillin‐binding protein 6a
*dacD*	K07258	D‐Alanyl‐D‐alanine carboxypeptidase; penicillin‐binding protein 6b
*dapA*	K01714	4‐Hydroxy‐tetrahydrodipicolinate synthase
*dapC (argD)*	K14267	N‐Succinyldiaminopimelate aminotransferase
*dapD*	K00674	2,3,4,5‐Tetrahydropyridine‐2‐carboxylate N‐succinyltransferase
*dapE (msgB)*	K01439	Succinyl‐diaminopimelate desuccinylase
*dapF*	K01778	Diaminopimelate epimerase
*ddl*	K01921	D‐Alanine‐D‐alanine ligase
*ftsA*	K03590	Cell division protein FtsA
*ftsB*	K05589	Cell division protein FtsB
*ftsE*	K09812	Cell division transport system ATP‐binding protein
*ftsI (pbpB)*	K03587	Transpeptidase involved in septal peptidoglycan synthesis (DD‐transpeptidase)
*ftsK (spoIIIE)*	K03466	DNA segregation ATPase
*ftsL (divIC)*	K03586	Cell division protein FtsL
*ftsN*	K03591	Cell division protein FtsN
*ftsP (sufl)*	K04753	Suppressor of FtsI
*ftsQ*	K03589	Cell division protein FtsQ
*ftsW (rodA, spoVE)*	K03588	Cell division protein FtsW, SEDS protein
*ftsX*	K09811	Cell division transport system permease protein
*ftsZ*	K03531	Cell division protein FtsZ (tubulin homolog)
*glmM*	K03431	Phosphoglucosamine mutase
*glmS*	K00820	Glucosamine‐‐fructose‐6‐phosphate aminotransferase
*glmU*	K04042	Bifunctional UDP‐N‐acetylglucosamine pyrophosphorylase / Glucosamine‐1‐phosphate N‐acetyltransferase
*glyA*	K00600	Glycine hydroxymethyltransferase, SHMT
*ldt (erfK, srfK)*	K16291	LD‐transpeptidase
*lpxT (yeiU)*	K19803	Lipid A 1‐diphosphate synthase; undecaprenyl pyrophosphate:lipid A 1‐phosphate phosphotransferase
*lysC (apk)*	K00928	Aspartate kinase
*metC*	K01760	Cystathionine beta‐lyase
*mepA*	K07261	Murein DD‐endopeptidase
*mepH*	K19303	Murein DD‐endopeptidase
*mepM*	K19304	Murein DD‐endopeptidase
*mepS*	K13694	Murein DD‐endopeptidase
*mltB*	K08305	Membrane‐bound lytic murein transglycosylase B
*mltC*	K08306	Membrane‐bound lytic murein transglycosylase C
*mltD*	K08307	Putative membrane‐bound lytic murein transglycosylase D
*mltE*	K08308	Lytic murein endotransglycosylase E
*mltF*	K18691	Membrane‐bound lytic transglycosylase F, murein hydrolase
*mltG*	K07082	Endolytic murein transglycosylase, septation protein, ampicillin sensitivity
*mraY*	K01000	Phospho‐N‐acetylmuramoyl‐pentapeptide‐transferase
*mrcA (ponA)*	K05366	Penicillin‐binding protein 1A/PBP1A (glycosyltransferase/DD‐transpeptidase)
*mrcB (ponB)*	K05365	Penicillin‐binding protein 1B/PBP1B (glycosyltransferase/DD‐transpeptidase)
*mrdA (pbpA)*	K05515	Penicillin‐binding protein 2/PBP2 (DD‐transpeptidase)
*mreB (mbl)*	K03569	Rod shape‐determining protein MreB and related proteins (actin homolog)
*mreC*	K03570	Rod shape‐determining protein MreC
*mreD*	K03571	Rod shape‐determining protein MreD
*mtgA*	K03814	Monofunctional glycosyltransferase
*murA (murZ)*	K00790	UDP‐N‐acetylglucosamine 1‐carboxyvinyltransferase
*murB*	K00075	UDP‐N‐acetylpyrovoylglucosamine dehydrogenase
*murC*	K01924	UDP‐N‐acetylmuramate‐L‐alanine ligase
*murD*	K01925	UDP‐N‐acetylmuramoylalanine‐‐D‐glutamate ligase
*murE*	K01928	UDP‐N‐acetylmuramoyl‐L‐alanyl‐D‐glutamate‐‐2,6‐diaminopimelate ligase
*murF*	K01929	UDP‐N‐acetylmuramoyl‐tripeptide‐‐D‐alanyl‐D‐alanine ligase
*murG*	K02563	UDP‐N‐acetylglucosamine‐‐N‐acetylmuramyl‐(pentapeptide) pyrophosphoryl‐undecaprenol N‐acetylglucosamine transferase
*murI (glr)*	K01776	Glutamate racemase
*murJ (mviN)*	K03980	Putative peptidoglycan lipid II flippase
*nagK*	K00884	N‐acetylglucosamine kinase
*nagZ*	K01207	Beta‐N‐acetylhexosaminidase
*pbpC*	K05367	Penicillin‐binding protein 1C (glycosyltransferase/DD‐transpeptidase)
*pbpG*	K07262	Serine‐type D‐Ala‐D‐Ala endopeptidase PBP7
*pgpB*	K01096	PAP2‐type phosphatidylglycerophosphatase / undecaprenyl‐diphosphate diphosphatase
*rodA (mrdB)*	K05837	Rod shape determining protein RodA, hypothetical lipid II flippase and/or glycosyltransferase
*rodZ (yfqA)*	K15539	Cytoskeleton‐associated protein
*slt (mltE)*	K08309	Soluble lytic murein transglycosylase
*uppS (yaeS)*	K00806	Undecaprenyl diphosphate synthase
*ybjG (bcrC)*	K19302	PAP2‐type undecaprenyl‐diphosphate diphosphatase

The unique lifecycle of obligate intracellular bacteria, while shielding them from extracellular innate immune surveillance, has resulted in particular selective pressures on their peptidoglycan, especially with respect to peptidoglycan‐sampling immune surveillance mechanisms (e.g., NOD1/2) located in the cytoplasm of host cells (Chaput and Boneca, 2007). Their location within the isotonic eukaryotic cell confers osmotic protection, whilst their constant proximity to host cell immune receptors means that they are under pressure to reduce recognition of this key PAMP. In this review, we explore the ability of obligate intracellular bacteria to assemble the peptidoglycan transiently during the cell cycle, at reduced levels and/or to synthesize a chemically modified version of peptidoglycan.

## The peptidoglycan of obligate intracellular bacteria

### Chlamydiales

The Chlamydiales are a large and diverse order of bacteria that include both human and animal pathogens, as well as non‐pathogenic environmental species. The two major human pathogens are *C. trachomatis* and *C. pneumoniae*. However, some of the lesser‐known so‐called ‘environmental’ chlamydia (sometimes referred to as ‘chlamydia‐like’ bacteria), which live inside amoeba, have recently been described as putative human pathogens, including *Waddlia chondrophila* and possibly *Simkania negevensis* (Ammerdorffer *et al*., 2017; Vouga *et al*., 2017). Chlamydiae are tropic for endothelial, epithelial and monocyte/macrophage cells and even primitive macrophage‐like cells such as amoebae (Kebbi‐Beghdadi and Greub, 2014). They secrete effector proteins through a type 3 secretion system to trigger dramatic rearrangement of the host cytoskeleton resulting in engulfment of the bacterium (Nans *et al*., 2015). Once phagocytosed by the host cell, Chlamydiae remodel the phagocytic vacuole, resulting in a specialised membrane‐surrounded organelle, called inclusion, that provides a protected environment for replication (Nans *et al*., 2015). The chlamydial cell type that is engulfed is the dispersal form known as the elementary body, a non‐replicative and poorly metabolically active cell type. Upon uptake, the elementary body differentiates within the inclusion into the replicative reticulate body which is no longer infectious (Nans *et al*., 2015). Chlamydiales are amongst the few known bacteria that lack FtsZ, the bacterial tubulin homolog that organizes the divisome complex at midcell to direct septal peptidoglycan synthesis and facilitates cytokinesis (Busiek and Margolin, 2015). Evidence has been provided that the bacterial actin complex, composed of MreB actin and its regulator RodZ, partially substitute for the role of FtsZ in cytokinesis and spatial regulation of septal peptidoglycan synthesis (Jacquier *et al*., 2014; Kemege *et al*., 2015; Liechti *et al*., 2016). After a complete replication cycle re‐differentiation into elementary bodies takes place in response to unknown signals, and new elementary bodies are released from the cell by either lysis or extrusion.

For many years, the chlamydial anomaly described the paradox of a non‐detectable peptidoglycan, despite the susceptibility of these organisms towards β‐lactams, which target peptidoglycan synthesis (Ghuysen and Goffin, 1999). Notably, unlike for other bacteria, β‐lactams are not bactericidal for chlamydia, but lead to a persistent infection of polyploid aberrant bodies (Skilton *et al*., 2009). The bactericidal action of β‐lactams in most bacteria is due to a lethal uncoupling of peptidoglycan synthetic and remodelling activities during growth and cell division, followed by lysis (Tomasz and Waks, 1975; Kohlrausch and Höltje, 1991; Cho *et al*., 2014). Although chlamydia can survive in the osmoprotective, intracellular environment, they cannot multiply without peptidoglycan synthesis, suggesting that peptidoglycan is required for chlamydial division *(*Henrichfreise *et al*., 2009; Skilton *et al*., 2009 *;* Jacquier *et al*., *2014;* 2015). The chlamydial anomaly has recently been resolved through the use of highly sensitive mass spectrometry techniques and newly developed fluorescent probes based on the peptidoglycan‐specific D‐Ala‐D‐Ala dipeptide (Liechti *et al*., 2013; Pilhofer *et al*., 2013). It is now known that several Chlamydiae possess peptidoglycan‐like structures, although the composition and arrangement seems to vary throughout the order. Complete peptidoglycan sacculi have been isolated and observed by cryoelectron tomography in some (but not all) environmental isolates (Pilhofer *et al*., 2013). However, the human pathogen *C. trachomatis* has no peptidoglycan sacculus but a discrete and transient peptidoglycan ring structure, which constricts together with the septum of dividing cells (Liechti *et al*., 2016; Packiam *et al*., 2015). Hence, even in the absence of a need for osmoprotection *C. trachomatis* cells maintain a rudimentary and β‐lactam‐sensitive peptidoglycan structure for cytokinesis. The elementary bodies of this species appears to maintain cell envelope integrity by a network of outer membrane proteins cross‐linked via disulphide bonds (Hatch *et al*., 1986).

*Chlamydia* are a member of the PVC superphylum (Planctomycetes‐Verrucomicrobia‐Chlamydiae). Whilst many of these genera are not obligate intracellular bacteria they will be briefly discussed here because they are unusual in having multiple members that are free living bacteria predicted to lack a peptidoglycan cell wall. The Verrucomicrobia, including *Akkermansia muciniphila*, possess a classical cell wall and are described as being diderm (Gram‐negative) species. In contrast, the Planctomycetes were long described as universally lacking peptidoglycan and having a proteinaceous cell wall instead. Two recent reports, however, have demonstrated the detection of a peptidoglycan‐like substance in some members of the planctomycetes (Jeske *et al*., 2015; van Teeseling *et al*., 2015). These include *Kuenenia stuttgartiensis Planctomyces limnophilus, Gemmata obscuriglobus* and *Rhodopirellula baltica*. Similar to the Chlamydiae, the peptidoglycan in these organisms was difficult to detect and present at low abundance.

### Rickettsiales

The order Rickettsiales contains two families: the Rickettsiaceae and the Anaplasmataceae (Eremeeva *et al*., 2005). Significant differences in their lifecycles and cell tropism may have resulted in distinct selective pressures on their peptidoglycan, and they will be discussed separately in this section. The order Rickettsiales is evolutionarily related to the predicted precursor of modern mitochondria (Emelyanov, 2001).

The Rickettsiaceae are a group of obligate intracellular, vector‐borne bacteria that cause a range of typhus‐like diseases in humans (Parola and Raoult, 2006; Walker, 2007). They primarily target the vascular endothelium, but *R. akari* is tropic to monocytes/macrophages (Radulovic *et al*., 2002) and *Orientia tsutsugamushi* is also found in monocytes/macrophages and dendritic cells (Moron *et al*., 2001; Paris *et al*., 2012). Unlike the Chlamydiales, the Rickettsiaceae cannot spread directly between infected individuals but are transferred via a mite, tick, louse or flea vector, likely due to a different tropism and/or poor survival outside cells compared to chlamydial elementary bodies. With the exception of *R. prowazekii*, which can spread directly between infected individuals via the body louse, most Rickettsiaceae are maintained through a range of animal reservoirs (Eremeeva and Dasch, 2015). Some Rickettsiaceae, such as *O. tsutsugamushi*, are also able to be transmitted transovarially to vector offspring, bypassing the absolute requirement for an intermediate animal reservoir (Shin *et al*., 2014; Takhampunya *et al*., 2016). Rickettsiaceae use a zipper‐like mechanism for uptake into the target cell (Ihn *et al*., 2000; Lee *et al*., 2008; Cho *et al*., 2010a). Once inside the cell, they escape from membrane‐enclosed vacuoles in the endolysosomal pathway and undergo growth and replication directly in the host cytosol (Chu *et al*., 2006). The bacteria are therefore directly exposed to autophagy machinery (Choi *et al*., 2013; Ko *et al*., 2013) and cytosolic immune receptors such as Nod1 and Nod2 (Cho *et al*., 2010b), and this has likely put selective pressure on these organisms to minimise receptor activation and downstream effectors.

With the exception of *Orientia*, which is a distinct genus within the Rickettsiaceae, the Rickettsiaceae are thought to possess a complete peptidoglycan structure and are sensitive to penicillin when grown in cultured cells (Silverman and Wisseman, 1978; Wisseman *et al*., 1982). *Orientia* is insensitive to ß‐lactams and was historically thought to lack any peptidoglycan structures (Amano *et al*., 1987), despite the presence of an apparently complete peptidoglycan biosynthetic pathway encoded in its genome (Cho *et al*., 2007; Min *et al*., 2008; Nakayama *et al*., 2008). Recent work has provided evidence that *O. tsutsugamushi* may possess a minimal peptidoglycan‐like structure and is sensitive to a range of non‐ß‐lactam peptidoglycan‐targeting antibiotics such as D‐cycloserine and phosphomycin, but remains insensitive to all ß‐lactams tested, which might be explained by an intrinsic insensitivity by the class B PBPs of this organism (Atwal *et al*., 2017) or an unknown beta‐lactamase. *O. tsutsugamushi* was also shown to possess a disulphide cross‐linked protein network on the outer membrane, analogous to the Chlamydiae (Atwal *et al*., 2017).

The Anaplasmataceae comprise a group of tick‐borne human and veterinary pathogens. This family also contains the nematode‐borne pathogen *Neorickettsia sennetsu* (Dittrich *et al*., 2015) and the prolific and promiscuous insect symbiont *Wolbachia* (Sicard *et al*., 2014). This family is unlike the Rickettsiaceae in primarily residing in erythrocytes, neutrophils and monocytes/macrophages and having a vacuolar cellular localisation (Carlyon and Fikrig, 2003; Rikihisa, 2003; Munderloh *et al*., 1999). Similar to the Chlamydiales, Anaplasmataceae exhibit a morphologically distinct biphasic life cycle, transitioning between non‐replicative and infectious dense‐core particles and replicating reticulate cells (Troese and Carlyon, 2009). *Anaplasma phagocytophilum* and *Ehrlichia chaffeensis* have been reported to lack both peptidoglycan and LPS, and this is supported by the absence of genes required for their synthesis (Lin and Rikihisa, 2003). Peptidoglycan has never been detected in *Wolbachia*, however, it has been shown that lipid II is essential for cell division (Vollmer *et al*., 2013) and *Wolbachia* contains a functional peptidoglycan amidase (Wilmes *et al*., 2017). There are no reports of the analysis or isolation of peptidoglycan in *Anaplasma marginale*. Anaplasmataceae have the ability to incorporate host‐derived lipids and sterols such as sphingolipids or cholesterol into their membranes, conferring some degree of structural rigidity in the absence of peptidoglycan (Lin and Rikihisa, 2003).

### Coxiella burnettii

*C. burnettii* is an intracellular γ‐proteobacterium and the causative agent of the human disease Q fever (van Schaik *et al*., 2013). It is generally described as an obligate intracellular bacterium, but specific growth media has recently been developed that supports growth in the absence of living host cells (Omsland *et al*., 2009). Similar to the Anaplasmataceae and *Chlamydiales, Coxiella* occupies a replicative niche within a membrane‐enclosed vacuole of an infected cell (Kohler and Roy, 2015). The *Coxiella‐*containing vacuole, however, is acidified, and this organism has developed mechanisms to survive and proliferate under these conditions (van Schaik *et al*., 2013).

*Coxiella* differentiates between the replicative large cell variant (LCV) form, and the non‐replicative short cell variant (SCV). Whilst both are capable of infecting cultured cells, the extracellular SCV form is the predominate agent of transmission in the environment. The SCV form of *Coxiella* is incredibly stable and able to withstand harsh environmental conditions such as extended desiccation and heat. It is highly infectious, with only 10 particles sufficient to cause disease in humans, making *Coxiella* a potential bioterrorism threat (Azad, 2007; Oyston and Davies, 2011).

Structural rigidity in SCV‐form *Coxiella* is partially conferred by a thick peptidoglycan, and it has recently been shown that this is characterised by an abundance of LD‐transpeptidase‐mediated 3‐3 peptide cross‐links (Sandoz *et al*., 2016).

### Mycobacterium leprae

*M. leprae* is a member of the Mycobacteriaceae family within the class Actinobacteria, and is the causative agent of the human disease leprosy (Rodrigues and Lockwood, 2011). Mycobacteria possess an almost impermeable, waxy cell surface with an unusual second membrane that is rich in mycolic acids and attached to a thick peptidoglycan layer via the polysaccharide arabinogalactan (Jankute *et al*., 2015; Nataraj *et al*., 2015). With 3.3 Mbp and 1,604 predicted proteins, *M. leprae* has a small genome compared with 4.4 Mbp and 3,924 predicted proteins in *M. tuberculosis* (Cole *et al*., 2001; Gutierrez *et al*., 2009), and extensive attempts to culture it in the laboratory have been unsuccessful (Lagier *et al*., 2015). It is therefore considered to be unique amongst mycobacteria in having an obligate intracellular lifecycle. *M. leprae* is predominantly localised in histiocytes and nerve cells, but is also found in macrophages and epithelial cells (Rodrigues and Lockwood, 2011). The peptidoglycan of *M. leprae* is comparable with that of other mycobacteria in that there is a high percentage of 3‐3‐cross‐links, but it lacks the N‐glycolation on muramic acid residues found in some mycobacteria, and it contains glycine in place of L‐alanines in a fraction of the peptides (Mahapatra *et al*., 2008).

### Buchnera

*Buchnera aphidicola* is the primary endosymbiont of the pea aphid *Acyrthosiphon pisum* (Douglas *et al*., 2011). Unlike the other obligate intracellular bacteria discussed here this organism is an obligate endosymbiont, and the insect host cannot survive in its absence. *Buchnera* is found within specialised polyploidy cells in the aphid body cavity, called bacteriocytes, where bacteria live within membrane‐enclosed vacuoles called symbiosomes. *Buchnera* is a γ‐proteobacterium, but lacks genes required for synthesising LPS in its outer membrane. Bacteria are spherical or oval in shape, are sensitive to penicillin and have a *meso*‐DAP‐containing peptidoglycan sacculus (Griffiths and Beck, 1974; Houk *et al*., 1977). Genome evolution of *B. aphidicola* has been intensively studied and it has been proposed to have evolved from an *E. coli*‐like genome by means of gene removal (Silva *et al*., 2001).

## Overview of this work

In the current study, we have sought to explore the relationship between the presence of enzymatic activities involved in peptidoglycan biosynthesis and turnover, and the presence of peptidoglycan in different obligate intracellular bacterial species. We have selected representatives of all major known groups of obligate intracellular bacteria, and have included a closely related free‐living organism as an outgroup for each species or group of species. This includes *C. crescentus* for the alpha‐proteobacteria (Rickettsiales); *E. coli* for the gamma‐proteobacteria (*Coxiella* and *Buchnera*); *B. subtilis* and *M. smegmatis* for the terrabacteria (*Mycobacterium leprae*) and *Akkermansia muciniphila* and *Planctomyces limnophilus* as planctomycetes and verrucomicrobia members of the PVC group respectively (Chlamydiae). These species were selected based on the availability of complete and annotated genomes and experimental evidence on the presence or absence of peptidoglycan (where available). We used a combination of KEGG database analysis and protein homology blast searches to identify proteins involved in different stages of peptidoglycan biosynthesis, and these are shown in Figs 4–7. Organisms are coloured according to their peptidoglycan status, with black indicating the complete absence of any peptidoglycan biosynthetic capacity (*Mycoplasma pneumoniae, Anaplasma phagocytophilum, Ehrlichia ruminatum*), red indicating a demonstrated low level or incomplete peptidoglycan sacculus (*Protochlamydia amoebophila, Chlamydia trachomatis, Planctomyces limnophilus* and *Orientia tsutsugamushi*) and green indicating the presence of a classical peptidoglycan sacculus (*Mycobacterium leprae, Mycobacterium smegmatis, Buchnera aphidicola, Rickettsia typhi, Caulobacter crescentus, Coxiella burnettii, Bacillus subtilis* and *Escherichia coli*). Where the peptidoglycan status is unknown, organisms are coloured in orange (*Wolbachia* strain *wMel, Anaplasma marginale*). In all dendrograms the organisms are grouped according to their similarity in protein profile across all genes considered in this study (Table 3 and full list shown in Supporting Information Table S1) and the proteins are grouped according to their similarity across all organisms considered in this work (Supporting Information Table S1).

## Pathways for peptidoglycan biosynthesis and remodelling

### Overview of peptidoglycan biosynthesis

**Figure 3:**
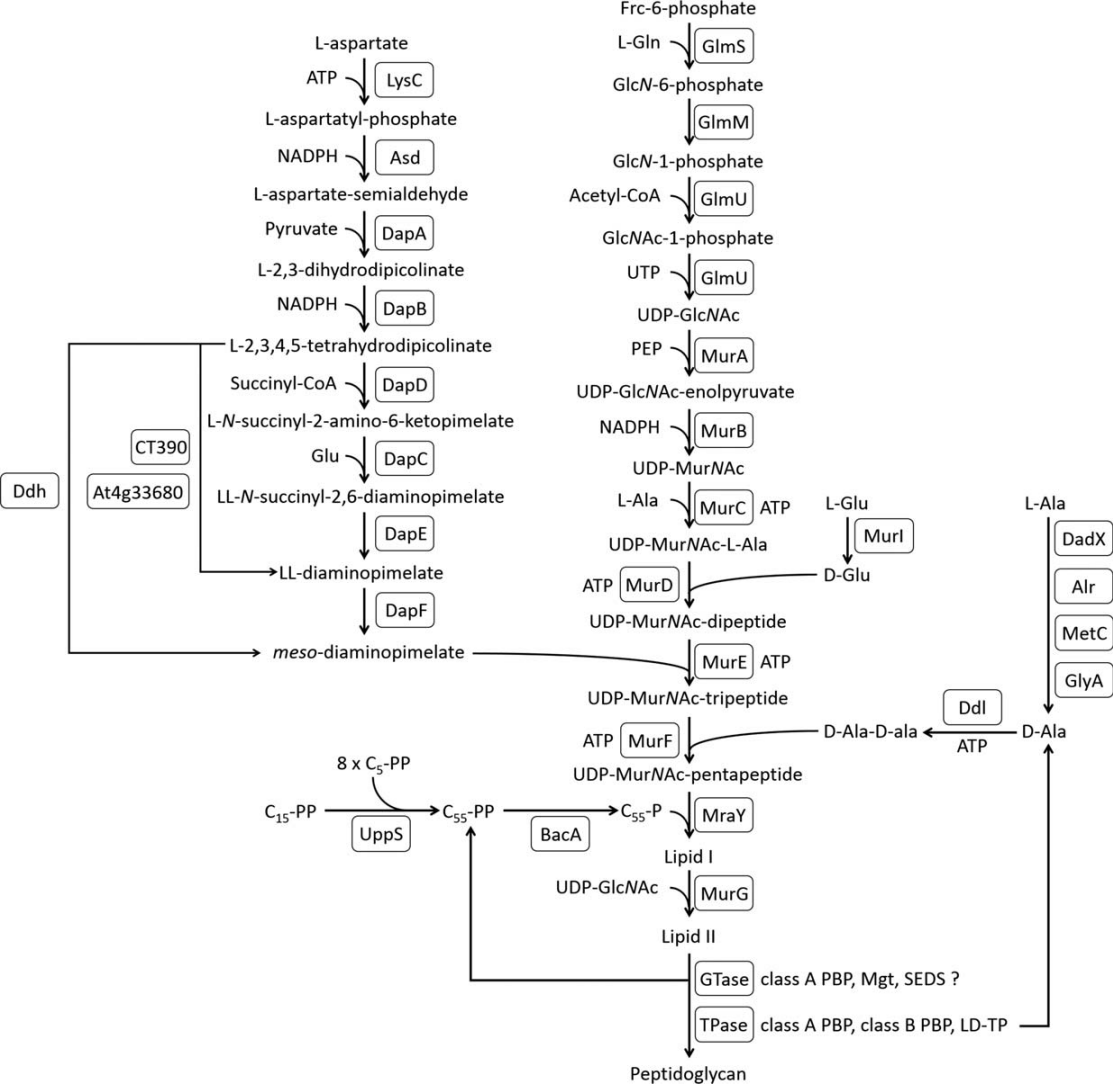
Peptidoglycan biosynthesis pathway. An overview of proteins involved in peptidoglycan biosynthesis. GTase, glycosyltransferase; TPase, transpeptidase; Mgt, monofunctional glycosyltransferase; LD‐TP, LD‐transpeptidase; SEDS, shape, elongation, division and sporulation; CT390, LL‐diaminopimelate aminotransferase from *Chlamydia trachomatis*; At4g33680, LL‐diaminopimelate aminotransferase from *Arabidopsis thaliana*; C_5_‐*PP*, isopentenyl‐pyrophosphate; C_15_‐*PP*, farnesyl‐pyrophosphate; C_55_‐*PP*, undecaprenyl‐pyrophosphate.

The peptidoglycan synthesis pathway starts in the cytoplasm with the formation of UDP‐MurNAc (Barreteau *et al*., 2008). Several further steps involving cytoplasmic amino acid racemaces, and D‐ and L‐amino acid ligases result in the synthesis of UDP‐MurNAc pentapeptide, which is the substrate for subsequent steps at the cytoplasmic membrane. MraY transfers MurNAc (pentapeptide) phosphate to the transport lipid undecaprenol phosphate (C55‐*P*) to form lipid I, and MurG catalyses the transfer of GlcNAc from UDP‐GlcNAc to lipid I to synthesize lipid II, the ultimate precursor for peptidoglycan synthesis (Bouhss *et al*., 2008). C55‐*P* is generated by UppS and a C55‐*PP* pyrophosphatase (BacA or PAP2 type). Lipid II is transported from the inner to the outer leaflet of the cytoplasmic membrane by a flippase, a process that remains an active area of research, with either SEDS proteins or MurJ being proposed as the lipid II flippase (Mohammadi *et al*., 2011; Sham *et al*., 2014). Lipid II is polymerized and the resulting nascent chains incorporated into the existing peptidoglycan layer by glycosyltransfer and transpeptidation reactions (Egan *et al*., 2015). These are catalysed by a group of membrane‐bound enzymes including penicillin‐binding proteins (PBPs), monofunctional glycosyltransferases and perhaps SEDS proteins (Meeske *et al*., 2016), and take place in the periplasm (Gram‐negative/diderm bacteria) or on the extracellular surface (Gram‐positive/monoderm bacteria) (Egan and Vollmer, 2013; Egan *et al*., 2017). The newly inserted peptidoglycan matures by the activities of synthetic and hydrolytic enzymes, such as LD‐transpeptidases and carboxypeptidases, and is eventually turned over by peptidoglycan hydrolases (amidases, endopeptidases, muramidases) during cell growth and division (Figs 3 and 7) (Höltje, 1998; Vollmer *et al*., 2008b). Some of the released peptidoglycan fragments are transported into the cytoplasm and trimmed for use in lipid II biosynthesis in a process called peptidoglycan recycling (Park and Uehara, 2008).

### Biosynthesis of peptidoglycan precursors

**Figure 4:**
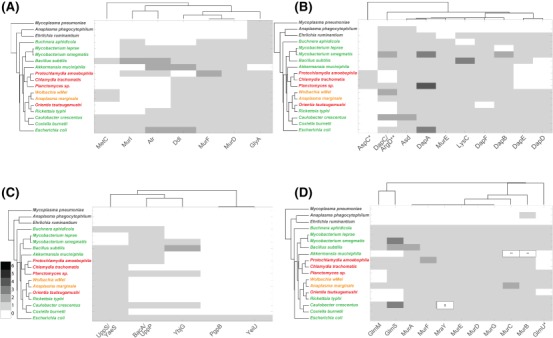
Peptidoglycan precursor enzymes. Dendograms showing the presence of proteins involved in different stages of PG biosynthesis in selected bacterial genomes. A. D‐amino acid generation and incorporation. B. meso‐DAP generation and incorporation. C. Generation and recycling of undecaprenyl phosphate. D. lipid II biosynthesis. The presence of multiple orthologs is indicated by colouring according to the key. The organism name is coloured according to peptidoglycan status: black, no peptidoglycan; red, intermediate or low‐level peptidoglycan; green, classical peptidoglycan sacculus; orange, peptidoglycan status unknown. The organisms are grouped according to the similarities in the presence/absence profiles for all proteins considered in this study (full list shown in Supporting Information Table S1) and the proteins are grouped according to their similarities across all organisms considered in this work. (B) *Including CT390 AT4G33680; **Merged profiles for K00821 and K14267. (D) *Merged profile for GlmU (K04042 and K11528); **Present as a fusion protein MurC‐MurB (ncbi id: ASB36453.1); § Genuine frameshift in *C. crescentus* CB15 but present and functional in NA1000.

The classical peptidoglycan precursor contains D‐Glu at position 2 and D‐Ala at positions 4 and 5 of the pentapeptide side chain (Vollmer *et al*., 2008a). Incorporation of D‐Glu and the D‐Ala‐D‐Ala dipeptide, which is synthesized by Ddl proteins, are catalysed by MurD and MurF respectively (Barreteau *et al*., 2008). The genes encoding these proteins are present across all organisms thought to possess peptidoglycan (Fig. 4A), although Ddl is present as a fusion protein with MurC in many Chlamydiae. The distribution of enzymes that catalyse amino acid racemisation reactions is more complicated. The classical amino acid racemases for these reactions are MurI, Alr and DadX. Whilst these are present in most of the organisms possessing classical peptidoglycan, their presence in intermediate peptidoglycan species is inconsistent. MetC has been shown to possess alternative alanine racemase activity in *E. coli* (Kang *et al*., 2011), and GlyA has been shown to be the alanine racemase in *Chlamydia pneumoniae* (De Benedetti *et al*., 2014), suggesting that the proteins used for racemase activity need not be strictly conserved in these pathways. It is also possible that Ddl, MurF and MurD may have different amino acid specificities in different organisms, and this can ultimately only be resolved by determining the structure of purified peptidoglycan from specific bacterial species.

Peptidoglycan from diderm bacteria typically contains *meso*‐DAP in position 3, which is linked to D‐Ala in position 4 of adjacent strands in mature peptidoglycan. MurE is the enzyme that mediates the incorporation of *meso*‐DAP into the peptidoglycan precursor (Barreteau *et al*., 2008) and this gene is present in all organisms that possess complete or intermediate peptidoglycan in this study (Fig. 4B). The distribution of *meso*‐DAP biosynthetic proteins is more complicated. DapD and DapE are missing in *P. amoebophila, C. trachomatis, Planctomycetes* sp. and *A. muciniphila*, but it has been shown that the LL‐diaminopimelate transferase (CT390) can perform the same reaction in *C. trachomatis* (McCoy *et al*., 2006)*. O. tsutsugamushi* lacks the enzyme catalysing the final step of *meso*‐DAP biosynthesis, DapF. Whilst no direct alternative to this enzyme has been described, a recent study showed the presence of *meso*‐DAP in purified *O. tsutsugamushi* by mass spectrometry, suggesting the presence of an unidentified alternative gene or pathway (Atwal *et al*., 2017). Surprisingly, *E. ruminantium* possesses a complete set of genes required for meso‐DAP biosynthesis, although it lacks MurE that would be required to incorporate it into the peptidoglycan precursor. This pathway is conserved in *E. chaffeensis* and *E. muris* (Supporting Information Table S1) and may have been retained during reductive evolution due to requirement of this metabolite in a different cellular pathway.

The MurNAc(‐pentapeptide) phosphate moiety from UDP‐MurNAc‐pentapeptide is transferred to the lipid anchor C55‐*P* to form lipid I. This bacterial polyisoprenoid anchors the building blocks for peptidoglycan and LPS synthesis to the inner leaflet of the membrane and enables flippases to shuttle them across the membrane. The MraY transferase synthesizing lipid I (Al‐Dabbagh *et al*., 2016) is present in all species that have complete or intermediate peptidoglycan, and absent in all those lacking peptidoglycan (Fig. 4D). The assembly of C55‐*PP* requires UppS (Manat *et al*., 2014), and its corresponding gene is encoded in almost all genomes of the peptidoglycan‐positive species in our study with the exception of *M. leprae* and *M. smegmatis* (Fig. 4C). In fact, *uppS* is absent in all mycobacteria (Supporting Information Table S1) and it is known that alternative, shorter lipid carriers can perform glycan transport in these organisms. The pyrophosphatases BacA (also called UppP), PgpB and YbjG (*E. coli*), required for the dephosphorylation of C55‐*PP* to C55‐*P* (Manat *et al*., 2014), were absent in many organisms in our study and this may reflect poor conservation of genes in the pathway.

MurG transfers GlcNAc from UDP‐GlcNAc to lipid I to form lipid II (Chen *et al*., 2002), which is transported across the membrane for polymerization (Fig. 3). Genes encoding MurA‐G and MraY are present in all organisms with complete and intermediate‐peptidoglycan, and are almost completely absent in those organisms lacking peptidoglycan (Fig. 4D). As expected, the *mur* genes are therefore a strong predictor of peptidoglycan status.

The generation of UDP‐GlcNAc is more problematic, with GlmM absent from some peptidoglycan‐intermediate species and GlmM and GlmS absent from all Rickettsiaceae and *A. muciniphila* (Fig. 4D and Supporting Information Table S1). It is possible that an alternative pathway is used for the generation of UDP‐GlcNAc, potentially employing novel importers.

### Growth of a peptidoglycan sacculus

Here, we discuss the membrane steps in peptidoglycan synthesis, including the flipping of lipid II across the cytoplasmic membrane, and the synthesis of cross‐linked peptidoglycan strands.

**Figure 5:**
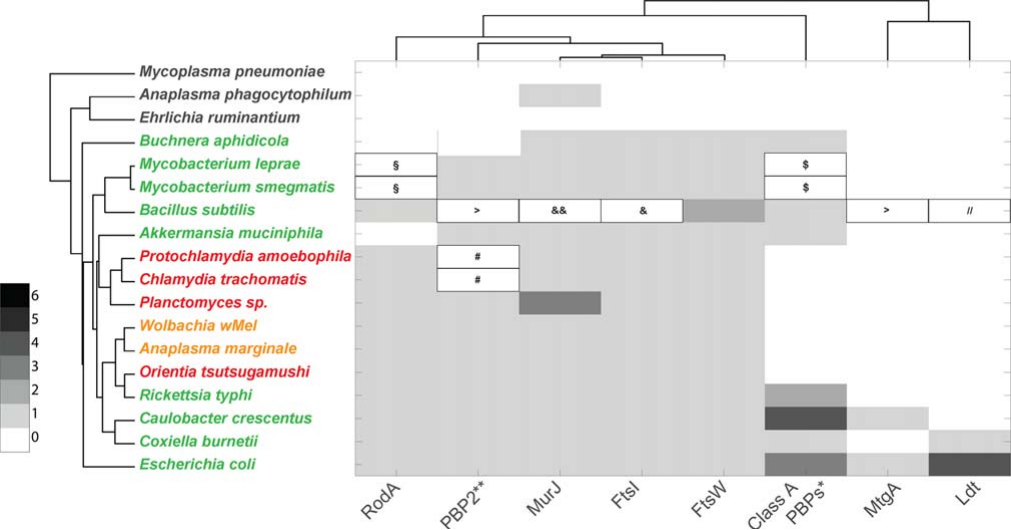
Dendogram showing the presence of peptidoglycan synthases, putative flippases, and SEDS proteins encoded by selected bacterial genomes. The organism name is coloured according to peptidoglycan status: black, no peptidoglycan; red, intermediate or low‐level peptidoglycan; green, classical peptidoglycan sacculus; orange, peptidoglycan status unknown. The organisms are grouped according to the similarities in the presence/absence profiles for all proteins considered in this study (full list shown in Supporting Information Table S1) and the proteins are grouped according to their similarities across all organisms considered in this work. The presence of multiple orthologs is indicated by colouring according to the key. *Class A PBPs contain merged profiles of MrcA (Kegg Accession Number KO5366, see also Supporting Information Table S1), MrcA2/PbpC (KO5367) and MrcB (KO5365). **PBP2 contains merged profiles of MrdA (KO5515) and PBPA (K05364). § Proteins annotated as RodA‐like are present (YP_884452 and NP_301145). # Protein NP_220201 is not present in Kegg as belonging to the PBP2 family, but it has been identified as such in Ouellette *et al*. (2012). The most similar sequence in *Protochlamydia amoebophila* is WP_011174685.1 (36% identical). $MSMEG_0031 and MLBr00018 are annotated as PbpA/PBP2 (K05364) they share < 30% identity with MrdA from *E. coli*. This ortholog group appears to be present in *Mycobacterium spp*. only (among the organisms considered in this work). & Protein annotated as FtsI‐PbpB is present (I33_1702), but it belongs to a different ortholog group in Kegg (K08724) && Protein similar to MurJ is present (I33_3060) assigned to no orthologous group.//Protein similar to Ldt proteins from *E. coli*, but not assigned to a K number in Kegg: I33_1583 (YkuD). > A protein belonging to ortholog group K21464 (PbpG) is present (I33_3896). This is also similar to MtgA from *E. coli*.

First, lipid II is transported from the inner to the outer leaflet of the cytosolic membrane to make it accessible for incorporation into existing peptidoglycan. The identity of lipid II flippase is an area of active research, with MurJ and SEDS proteins (FtsW, RodA) being primary candidates (Ruiz, 2008; Mohammadi *et al*., 2014, 2011; Sham *et al*., 2014). At least one copy of MurJ, FtsW and RodA‐like proteins were present in all peptidoglycan‐positive and peptidoglycan‐intermediate species in our analysis, with the exception of *Buchnera* and *A. muciniphila* which lack RodA, and whilst FtsW was absent from all peptidoglycan‐negative species MurJ was found in *A. phagocytophilum* (Fig. 5).

Following flippase activity, two specific enzymatic activities are required to incorporate lipid II into an extended peptidoglycan structure. Glycosyltransferase activity polymerizes the glycan strands and transpeptidase activity cross‐links peptides from adjacent glycan strands. The most common cross‐links are DD‐type, between D‐Ala in position 4 of one peptide and meso‐DAP in position 3 of another, but they can also be of the LD‐type which forms between two meso‐DAP residues (Höltje, 1998; Vollmer *et al*., 2008a). Glycosyltransferase activity in *E. coli* is largely performed by class A bifunctional PBPs, which possess both glycosyltransferase and transpeptidase activity, and a monofunctional glycosyltransferase (MtgA) (Egan *et al*., 2015). It was recently suggested that RodA from *Bacillus subtilis* also possesses glycosyltransferase activity, and it was hypothesised that this may be a general property of SEDS proteins and a possible source of glycosyltransferase activity in organisms lacking class A PBPs and monofunctional glycosyltransferases (Meeske *et al*., 2016). However, the *E. coli* SEDS protein FtsW lacked glycosyltransferase activity and instead controlled the glycosyltransferase activity of PBP1B in the presence of the class B PBP3 (Leclercq *et al*., 2017). DD‐transpeptidase activity is performed by class A PBPs (*E. coli* PBP1A, PBP1B, PBP1C) and class B PBPs (*E. coli* PBP2, PBP3), LD‐transpeptidase activity for the formation of 33‐cross‐links is performed by LD‐transpeptidases (*E. coli* YcbB, YnhG) (Magnet *et al*., 2008).

In our bioinformatics analysis of transpeptidase and glycosyltransferase activity (Fig. 5), we found that class A PBPs are absent from all the bacteria with intermediate peptidoglycan, but that representatives of both class B PBPs and SEDS proteins are present. Assuming that the transient peptidoglycan of these organisms contains glycan strands as for members of the chlamydia group (Pilhofer *et al*., 2013; Packiam *et al*., 2015), then the required glycosyltransferase activity comes either from the SEDS protein(s) or from a yet unknown glycosyltransferase. LD‐transpeptidases are present in *E. coli, B. subtilis* and *C. burnettii* but could not be identified in any of the other obligate intracellular organisms that we analysed, based on classifications provided by the Kegg database.

### Peptidoglycan trimming, degradation and recycling

**Figure 6:**
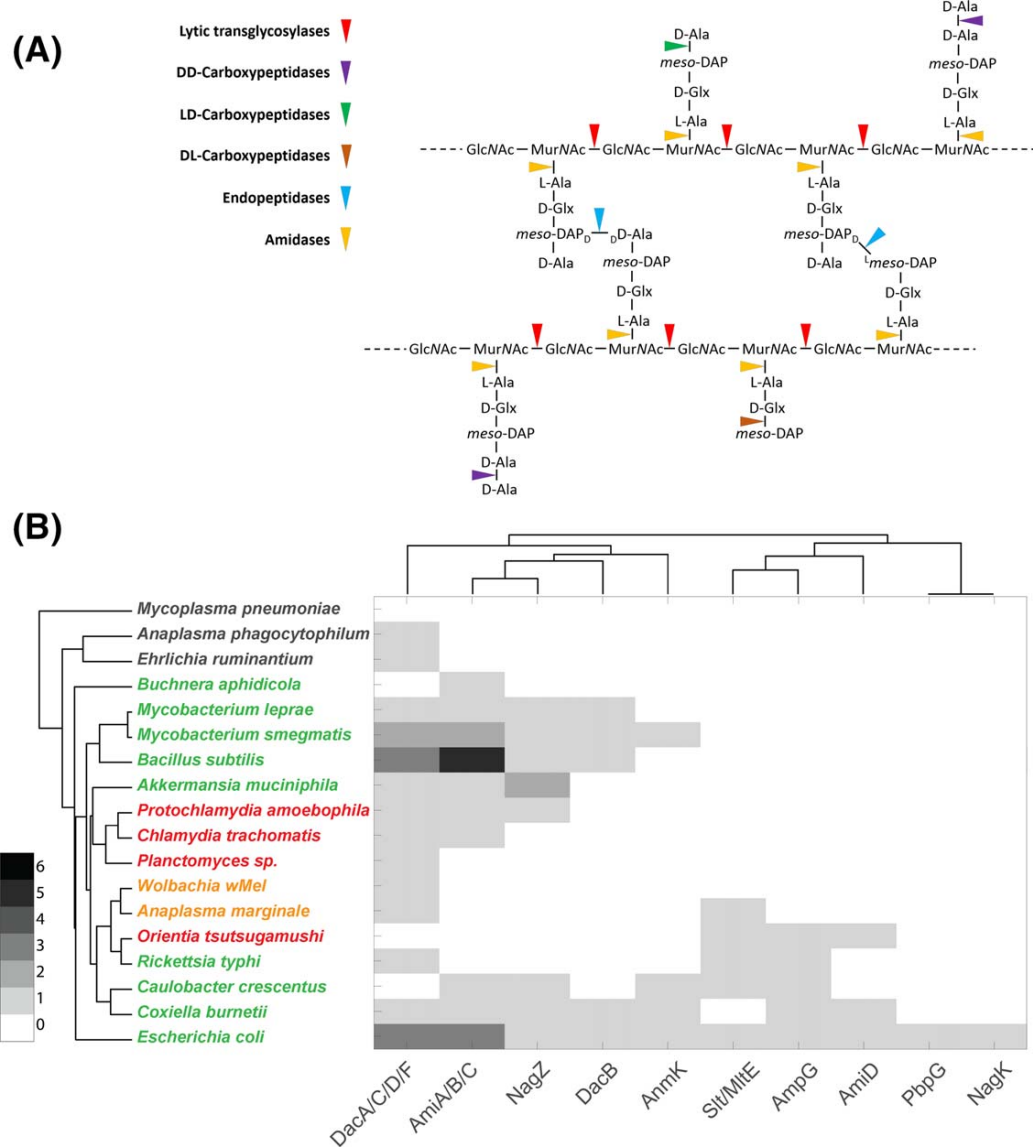
Peptidoglycan degradation and recycling. A. Overview of enzymes involved in peptidoglycan degradation. B. Dendogram showing the presence of peptidoglycan degradation and recycling proteins encoded by selected bacterial genomes. The organism name is coloured according to peptidoglycan status: black, no peptidoglycan; red, intermediate or low‐level peptidoglycan; green, classical peptidoglycan sacculus; orange, peptidoglycan status unknown. The organisms are grouped according to the similarities in the presence/absence profiles for all proteins considered in this study (full list shown in Supporting Information Table S1) and the proteins are grouped according to their similarities across all organisms considered in this work. The presence of multiple orthologs is indicated by colouring according to the key.

Nascent or polymerised peptidoglycan is often subject to secondary modifications, such as N‐deacetylation of residues in glycan chains, and trimming of the peptides by carboxypeptidases (Vollmer, 2008; Peters *et al*., 2016). Other peptidoglycan hydrolases cleave at various sites in the glycan and peptide components of peptidoglycan and these activities cause a release of peptidoglycan fragments from the sacculus (peptidoglycan turnover) (Vollmer *et al*., 2008b). Peptidoglycan hydrolases are a large and diverse group of enzymes, whose cleavage sites are shown in Fig. 6A. The released fragments are partly released into the extracellular space, and partly taken up and recycled into new peptidoglycan precursors (Park and Uehara, 2008; Borisova *et al*., 2016). The release of extracellular peptidoglycan is immunogenic and it is likely that obligate intracellular bacteria would limit the release of these molecules. Our bioinformatics analysis of peptidoglycan degradation and recycling genes shows a diverse pattern of genes in the selected organisms (Fig. 6B). The DD‐carboxypeptidase proteins DacA, DacC, DacD and DacF are present in many peptidoglycan‐positive and peptidoglycan‐negative organisms and may have another role in addition to peptidoglycan degradation. There was no consistent pattern in the distribution of hydrolase activities in the intermediate‐peptidoglycan group. *Orientia* and *A. marginale* genomes do not encode an AmiA/B/C‐like amidase, in common with all Rickettsiales, but possess a lytic transglycosylase (MltE) homologue. In contrast, lytic transglycosylase genes are absent in peptidoglycan‐negative *A. phagocytophilum* and *E. ruminantium* and obvious lytic transglycosylase‐like genes were not detected in the chlamydial genomes either, raising the possibility that additional enzymes can cleave glycan strands. Interestingly, AmiA from *C. pneumoniae* showed not only amidase but also DD‐carboxypeptidase activity (Klöckner *et al*., 2014). The latter, unexpected activity could be attributed to two motifs usually found in PBPs and was inhibited by β‐lactams.

**Figure 7:**
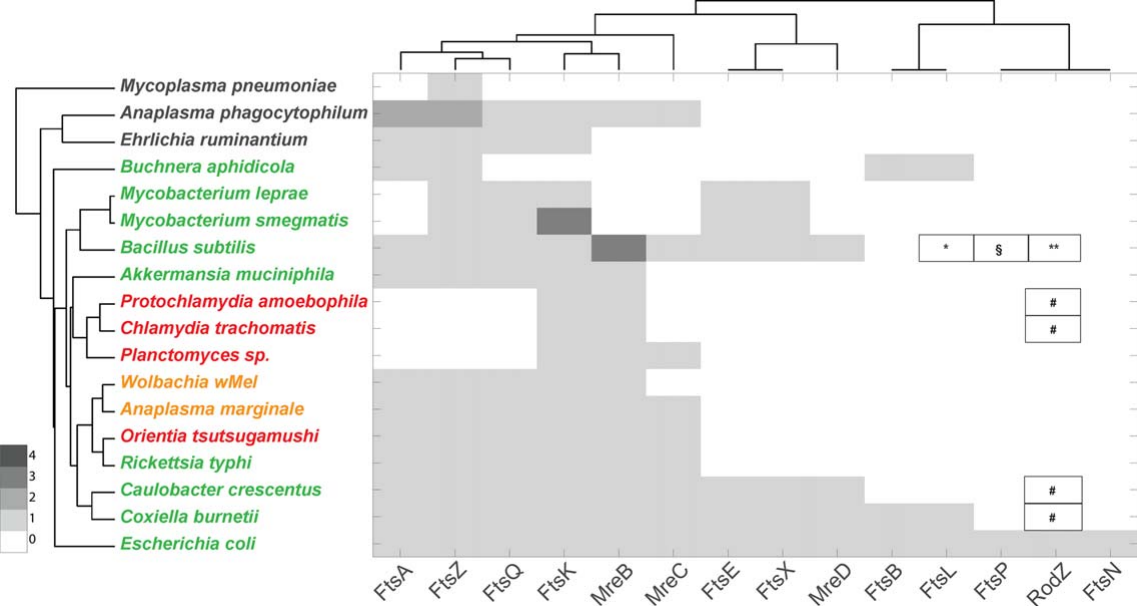
Dendogram showing the presence of cell morphogenesis proteins encoded by selected bacterial genomes. The organism name is coloured according to peptidoglycan status: black, no peptidoglycan; red, intermediate or low‐level peptidoglycan; green, classical peptidoglycan sacculus; orange, peptidoglycan status unknown. The organisms are grouped according to the similarities in the presence/absence profiles for all proteins considered in this study (full list shown in Supporting Information Table S1) and the proteins are grouped according to their similarities across all organisms considered in this work. The presence of multiple orthologs is indicated by colouring according to the key. # Proteins annotated to this function are present in the genomes of these species, *Coxiella burnetii* NP_820244.1; *C. crescentus* ADW96154.1; *Chlamydia trachomatis* NP_219511.1; *Protochlamydia amoebophila* CAF23404. *I33_1701 is annotated as FtsL but it is not included in any ortholog group. **Orthologs of I33_1877 in other Bacillus species are annotated as RodZ, but these sequences are not included in the RodZ ortholog group in Kegg. § A Multicopper oxidase with three cupredoxin domains (includes cell division protein FtsP and spore coat protein CotA) is present in strain 168 (BSU06300) but not in RO‐NN‐1

### Cell morphogenesis proteins

The peptidoglycan biosynthesis machinery is positioned and/or regulated through association with a network of cytosolic and membrane‐bound proteins involved in bacterial growth and morphogenesis (Egan *et al*., 2017). Two major components of the bacterial cytoskeleton are the tubulin homolog FtsZ and the actin homolog MreB (Ouellette *et al*., 2012; Celler *et al*., 2013). Both have been shown to generate dynamic filaments that are associated with PBPs and to guide the incorporation of nascent peptidoglycan (Dominguez‐Escobar *et al*., 2011; Garner *et al*., 2011). Whilst the Chlamydiae lack FtsZ (Frandi *et al*., 2014), MreB is absent in *Buchnera* and mycobacteria (Letek *et al*., 2008) (as well as the peptidoglycan‐negative mycoplasma and ehrlichiae). This underscores the fact that cytoskeleton‐guided peptidoglycan incorporation may be conserved in bacteria, but that there are different combinations of cytoskeletal elements and peptidoglycan synthesis enzymes across the kingdom, presumably reflecting unique aspects of the cell division apparatus and modes of peptidoglycan synthesis in different organisms.

### Predicting the peptidoglycan status of obligate intracellular bacteria

**Figure 8:**
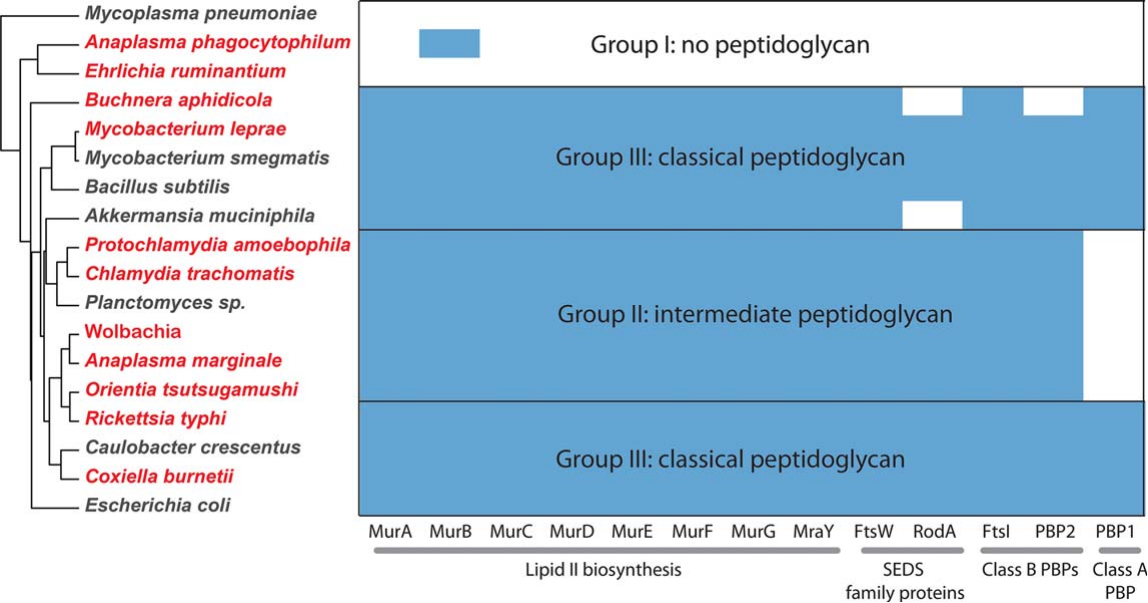
Summary of the presence/absence of key peptidoglycan biosynthesis gene homologs together with predictions about corresponding PG status. Obligate intracellular bacteria are shown in red, and free‐living bacteria are shown in black.

A small number of genes were highly correlated with the predicted peptidoglycan status, and these are summarised in Fig. 8. We observed that those bacteria with demonstrated low level of peptidoglycan or an incomplete sacculus were associated with a signature gene set: the presence of lipid II biosynthesis genes *murA*‐*murG* and *mraY*; the presence of at least one SEDS gene *ftsW* or *rodA*; the presence of at least one gene encoding a class B monofunctional PBP, but the notable absence of any detectable genes for class A bifunctional PBPs. Based on this classification we would predict that *Wolbachia* and *Anaplasma marginale* both possess some sort of peptidoglycan‐like structure, and we term this group ‘peptidoglycan‐intermediate’ organisms.

## Discussion

In this review, we explored the relationship between the distribution of genes involved in various aspects of cell wall biology, and the intracellular replicative niche adopted by obligate intracellular bacteria. We hypothesise that the selective pressures of an osmoprotective environment, combined with proximity to cellular host immune responses, would lead organisms to reduce their levels of peptidoglycan. Indeed, we observed common patterns of gene loss and retention in groups of unrelated obligate intracellular bacteria (Fig. 8). We termed this group peptidoglycan‐intermediate. This group was characterised by the presence of genes encoding orthologs of MurA‐MurG, MraY, SEDS proteins RodA/FtsW and the class B PBPs PBP2/PBP3(FtsI), but the notable absence of any class A PBPs. This group of organisms included pathogenic and environmental Chlamydiae as well as *Orientia tsutsugamushi, Wolbachia* strain *wMel* and *Anaplasma marginale*. Within this group the detailed structure of peptidoglycan determined by mass spectrometry is only known for *Chlamydia trachomatis* and it will be interesting to see how conserved patterns of gene retention translates to commonalities and differences in peptidoglycan structure across this group. It is expected that there will remain substantial differences in structure and arrangement, since it is already known that the peptidoglycan of *Protochlamydia amoebophila* forms a complete sacculus but peptidoglycan of *Chlamydia trachomatis* has only been detected as a discrete ring located at the septum and no peptidoglycan sacculus could be isolated from the chlamydia‐like bacterium *Simkania negevensis*. There are also likely to be lineage‐specific differences in the composition of peptidoglycan, for example unidentified chemical modifications have been shown for *Protochlamydia amoebophila* (Pilhofer *et al*., 2013) and there is some circumstantial evidence consistent with a modification of muropeptides after antibiotic treatment in *Chlamydia trachomatis* (Packiam *et al*., 2015). Species‐specific variations in peptidoglycan structure and composition might therefore reflect unique aspects of the individual cell biology and the host‐pathogen interactions of each obligate intracellular lineage.

The classification of an intermediate peptidoglycan group raises questions about why different closely related obligate intracellular bacteria would adopt different peptidoglycan statuses. For example, whilst all *Rickettsia* encode genes for a classical peptidoglycan sacculus, the sister genus *Orientia* lacks class A PBPs and was classified as peptidoglycan‐intermediate. The pressure for a reduced peptidoglycan structure in *Orientia* is unlikely to result solely from vector difference (many *Rickettsia* are tick‐borne whilst *Orientia* is mite‐borne) because *Rickettsia akari* is also mite‐borne and possesses complete peptidoglycan genes in common with other *Rickettsia. Orientia* is associated with dendritic cells and monocytes/macrophages in addition to endothelial cells in human patient eschar tissue samples, in contrast to most *Rickettsia*, which are predominantly endothelial‐cell localised. It is possible that this has led to a specific selective pressure on *Orientia* to reduce peptidoglycan, although it is worth noting that *Rickettsia akari* also localises in monocyte/macrophage cells. In common with this line of reasoning, it is notable that *A. marginale* possesses a complement of genes supporting the production of intermediate peptidoglycan, whilst the closely related *A. phagocytophilum* completely lacks the ability to produce peptidoglycan. This may also reflect differences in cell tropism since *A. marginale* is localised in erythrocytes whilst *A. phagocytophilum* is localised in monocytes/macrophages, potentially resulting in a difference in immune‐driven selective pressure on peptidoglycan status. Lastly, it is also possible that species‐specific chemical modifications exist that influence the magnitude of the signalling response of peptidoglycan innate immune surveillance systems.

The total lack of peptidoglycan in some organisms, as well as the presence of only intermediate peptidoglycan in others raises the question of how bacterial cells are structured in the absence of a complete classical peptidoglycan sacculus. *Mycoplasma* is unusual in lacking peptidoglycan but not being an obligate intracellular bacterium. It can replicate both in extracellular tissue fluid as well as within eukaryotic cells, and this lifestyle confers osmotic protection as well as a source of sterol lipids that are essential for their growth and likely protects the cell from rupture in the absence of a rigid cell wall. Host‐derived lipids are also important features of the membranes of Anaplasmataceae, conferring structural rigidity in the absence of a cell wall. Both Chlamydiae and *Orientia* possess disulphide cross‐linked proteins on their outer membranes and these confer additional structural rigidity in the absence of a full peptidoglycan cell wall sacculus. It is also conceivable that the proliferation mode can shape the genomic repertoire of peptidoglycan biosynthesis and remodelling genes. For example although the peptidoglycan‐intermediate *Planctomycetes* are free‐living bacteria, they reproduce by budding rather than binary fission typically executed by most other bacteria.

In conclusion, our analyses suggest that a group of diverse obligate intracellular bacteria have responded to selective pressures imposed by this lifestyle by selectively retaining a subset of genes in the peptidoglycan‐biosynthesis pathway. We hypothesise that this will result in some commonalities in peptidoglycan structure, which may include reduced overall abundance, differences in chain length and peptide cross linking, and regulated or limited biosynthesis in time and space. Future structural and biochemical analyses of the peptidoglycan from this group will lead to a greater understanding of the relationship between bacterial cell growth and host immune recognition, as well as commonalities of a minimal peptidoglycan cell wall, in this unique group of bacterial organisms.

## Supporting information

Supporting Table

Supporting Information
