# Supplementary material for: Peptidoglycan in obligate intracellular bacteria
Source: Mol Microbiol. 2017 Dec 12;107(2):142–63. doi: 10.1111/mmi.13880 (PMC5814848; doi:10.1111/mmi.13880)
Supplement: Supplementary file 2 — Supporting Information [file MMI-107-142-s002.pdf]

Peptidoglycan in Obligate Intracellular Bacteria

Supplementary Materials

Christian Otten[a]*, Matteo Brilli[b]*, Waldemar Vollmer[a], Patrick H. Viollier[c] and Jeanne Salje[d,e#]

Running title: Peptidoglycan in obligate intracellular bacteria

[a] The Centre for Bacterial Cell Biology, Institute for Cell and Molecular Biosciences, Newcastle University, Newcastle upon Tyne, NE2 4AX, United Kingdom

[b] DAFNAE, Department of Agronomy, Food, Natural Resources, Animals and Environment, University of Padova. Agripolis - V.le dell'Università, 16 | 35020 Legnaro (PD), Italy. Present address: Department of Biosciences , University of Milan, via Celoria 26, Milan (MI), Italy.

[c] Dept. Microbiology and Molecular Medicine, Institute of Genetics & Genomics in Geneva (iGE3), Faculty of Medicine, University of Geneva, Geneva, Switzerland

[d] Centre for Tropical Medicine and Global Health, Nuffield Department of Medicine, University of Oxford, Oxford, United Kingdom

[e] Mahidol-Oxford Tropical Medicine Research Unit, Faculty of Tropical Medicine, Mahidol University, Bangkok, Thailand

*These authors contributed equally

[#]Correspondence to Jeanne Salje: jeanne.salje@ndm.ox.ac.uk

Materials and Methods

1. Construction of species phylogenetic trees

Ortholog sequences corresponding to the GyrB protein to be used for phylogenetic analyses were retrieved from KEGG; the alignment was generated using Muscle and default parameters for protein sequences, translated into phylip format and loaded in PhyML (Guindon et al., 2010). The automatic selection of the best evolutionary model indicated the LG model as the most suitable for this dataset and was used for phylogenetic reconstruction together with fast likelihood-based method for branch support based on a Bayesian-like transformation of aLRT (Anisimova et al., 2011). Evolutionary rates were allowed to vary among sites in the multi-alignment and were approximated with a discrete Gamma distribution with 4 categories.

2. Generation of phylogenetic profile dendograms

Phylogenetic profile reconstructions were performed by exploiting ortholog tables available in Kegg (Kanehisa, M. and Goto, S. 2000). Scripts were developed to build presence/absence matrices starting from a number of reference proteins to retrieve the corresponding ortholog group and check for presence in our genome dataset. Phylogenetic profiles were amended when experimental evidences were in disagreement with the computed profiles. Dendrograms in Figures N-M were built using the jaccard distance on binarized phylogenetic profiles. The dendrogram of organisms was built considering all the proteins retrieved in this work (Supplemental Table 1), even if in the

figures we only show a subset of them. Similarly, for each group of proteins in the figures, the dendrogram was obtained by considering the entire set of organisms in our starting dataset. In this way the clustering is much more robust than using the datasets corresponding to each figure, separately.

References:

Anisimova M., Gil M., Dufayard JF., Dessimoz C. and Gascuel O.; Survey of branch support methods demonstrates accuracy, power, and robustness of fast likelihood-based approximation schemes. Systematic Biology, 60(5):685-99 (2011)

Guindon S., Dufayard J.F., Lefort V., Anisimova M., Hordijk W., Gascuel O.; New Algorithms and Methods to Estimate Maximum-Likelihood Phylogenies: Assessing the Performance of PhyML 3.0. Systematic Biology, 59(3):307-21 (2010)

Kanehisa, M. and Goto, S.; KEGG: Kyoto Encyclopedia of Genes and Genomes. Nucleic Acids Res. 28, 27-30 (2000).

Stamatakis, A: "RAxML Version 8: A tool for Phylogenetic Analysis and Post-Analysis of Large Phylogenies". In Bioinformatics, 2014

Supplementary Table 1

A complete list of all genes retrieved in our bioinformatics analysis with embedded links to the Kegg database entry for each gene. Available as a separate supplementary file.
